# Mechanistic Studies of Anti-Hyperpigmentary Compounds: Elucidating Their Inhibitory and Regulatory Actions

**DOI:** 10.3390/ijms150814649

**Published:** 2014-08-21

**Authors:** Rosanna Y. Y. Lam, Zhi-Xiu Lin, Elena V. Sviderskaya, Christopher H. K. Cheng

**Affiliations:** 1 School of Chinese Medicine, Faculty of Medicine, The Chinese University of Hong Kong, Shatin, N.T., Hong Kong, China; E-Mails: rorolam@hotmail.com (R.Y.Y.L.); linzx@cuhk.edu.hk (Z.-X.L.); 2 Faculty of Medicine and Biomedical Sciences, St. George’s, University of London, Cranmer Terrace, London SW17 0RE, UK; E-Mail: esviders@sgul.ac.uk; 3 School of Biomedical Sciences, Faculty of Medicine, The Chinese University of Hong Kong, Shatin, N.T., Hong Kong, China; 4 Centre of Novel Functional Molecules, The Chinese University of Hong Kong, Shatin, N.T., Hong Kong, China

**Keywords:** depigmenting agents, hyperpigmentation, melan-a, 4-ethylresorcinol, 4-ethylphenol, 1-tetradecanol

## Abstract

Searching for depigmenting agents from natural sources has become a new direction in the cosmetic industry as natural products are generally perceived as relatively safer. In our previous study, selected Chinese medicines traditionally used to treat hyperpigmentation were tested for anti-hyperpigmentary effects using a melan-a cell culture model. Among the tested chemical compounds, 4-ethylresorcinol, 4-ethylphenol and 1-tetradecanol were found to possess hypopigmentary effects. Western blot analysis, reverse transcriptase polymerase chain reaction (RT-PCR), cyclic adenosine monophosphate (cAMP) assay, protein kinase A (PKA) activity assay, tyrosinase inhibition assay and lipid peroxidation inhibition assay were performed to reveal the underlying cellular and molecular mechanisms of the hypopigmentary effects. 4-Ethylresorcinol and 4-ethylphenol attenuated mRNA and protein expression of tyrosinase-related protein (TRP)-2, and possessed antioxidative effect by inhibiting lipid peroxidation. 1-Tetradecanol was able to attenuate protein expression of tyrosinase. The hypopigmentary actions of 4-ethylresorcinol, 4-ethylphenol and 1-tetradecanol were associated with regulating downstream proteins along the PKA pathway. 4-Ethylresorcinol was more effective in inhibiting melanin synthesis when compared to 4-ethylphenol and 1-tetradecanol.

## 1. Introduction

Melanogenesis forms a natural protective system against UV-induced damage on the skin. However, over production of melanin can lead to hyperpigmentary disorders such as freckles, chloasma and lentigines. Hyperpigmentation is one of the most common cosmetic problems affecting many people around the world and the demands for skin whitening agents to combat hyperpigmantary disorders remain substantial.

Melanogenesis is a complex biochemical process involving a number of cell signaling pathways. To understand the pharmacological mechanisms of potential anti-hyperpigmentary compounds, it is necessary to study their effects on the signaling pathways within the melanocytes, and the cross-talk of those pathways. The most frequently studied melanogenic pathways include protein kinase A (PKA), p38 mitogen-activated protein kinase (MAPK), phosphatidylinositol 3-kinase (PI3K) and extracellular signal-regulated kinase (ERK) pathways.

In the PKA cascade, cyclic adenosine monophosphate (cAMP), which is converted from adenosine triphosphate, first stimulates PKA [1]. Activated PKA then phosphorylates and activates cAMP response element binding protein (CREB) which in turn binds to the cAMP response element located at the M promoter of the microphthalmia-associated transcription factor (*MITF*) gene [2]. The elevation of MITF expression stimulates the transcription and translation of tyrosinase, tyrosinase-related protein (TRP)-1 and TRP-2 [3]. These three proteins are localized in the melanosomal membrane [4]. Their activation promotes the onset of melanogenesis. Tyrosinase is the rate-limiting enzyme [4,5] and participates in the first reaction step of melanogenesis in the melanosome which catalyses tyrosine hydroxylation. It also catalyses 3,4-dihydroxyphenylalanine (DOPA) dehydrogenation and 5,6-dihydroxyindole dehydrogenation [6]. TRP-1 acts as a 5,6-dihydroxyindole-2-carboxylic acid (DHICA) oxidase which converts DHICA to indole-quinones [7,8,9]. TRP-2 acts as a dopachrome tautomerase that catalyses the tautomerization of dopachrome to DHICA [10,11].

In the p38 MAPK cascade, active PKA stimulates p38 MAPK, then p38 MAPK phosphorylates and activates MITF [12,13], subsequently initiating melanogenesis. On the other hand, in the PI3K cascade, cAMP inhibits PI3K and Akt [14,15], then glycogen synthase kinase (GSK) 3β is activated [16]. GSK 3β phosphorylates MITF and enhances the binding of MITF to the M-box of the tyrosinase promoter, thereby stimulating tyrosinase expression [17]. GSK 3β also phosphorylates Tau [18]. Furthermore, in the ERK cascade, cAMP activates Ras, Raf, MAPK kinase (MEK), ERK and then ribosomal S6 kinase (RSK) [19,20,21]. ERK and RSK initiate the degradation of MITF, resulting in the attenuation of melanogenesis [22,23,24,25]. The pathway acts as a feedback mechanism to avoid melanin over production [21].

A number of hypopigmentary therapies are now available for hyperpigmentary disorders including laser therapy, plastic surgery and whitening chemical agents. An ideal hypopigmentary agent should have the ability to inhibit the activity of the rate-limiting enzyme tyrosinase and/or attenuate the expression of any proteins in the respective melanogenic pathways. Therefore, compounds that exert potent depigmenting effect at both enzymatic and cellular levels continue to be investigated. Searching for effective hypopigmentary compounds from natural sources is a hot pursuit in the cosmetic industry since natural products are generally perceived by consumers as free from, or having few side-effects.

**Figure 1:**
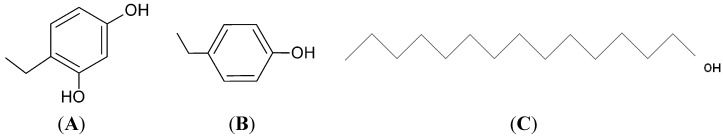
The chemical structures of (**A**) 4-ethylresorcinol; (**B**) 4-ethylphenol; and (**C**) 1-tetradecanol which elicit anti-hyperpigmentary effect in melan-a cells.

In our previous study, we employed melan-a, a non-tumorigenic cell line, as an* in vitro* system to screen a number of selected Chinese herbs for their potential inhibition on melanogenesis. We also reported the development and validation of an assay which involves the combination of sulphorhodamine B (SRB) and melanin assays, and the application of this combined assay on the screening of herbal extracts and natural compounds for hypopigmentary agents [26]. Among the tested chemical compounds, 4-ethylresorcinol, 4-ethylphenol and 1-tetradecanol (Figure 1) were found to elicit inhibitory effect on melanin synthesis in melan-a cells. As a continuation of our endeavors to develop hypopigmentary pharmaceutical agents, in the present follow-up study, we aimed to evaluate the underlying biochemical mechanisms of these effective compounds using a panel of biochemical assays including Western blot analysis, reverse transcriptase polymerase chain reaction (RT-PCR), cAMP assay, PKA activity assay, tyrosinase inhibition assay and lipid peroxidation inhibition assay.

## 2. Results

### 2.1. Effects of the Chemical Compounds on Signaling Protein Expression Levels in Melan-a Cells

**Figure 2:**
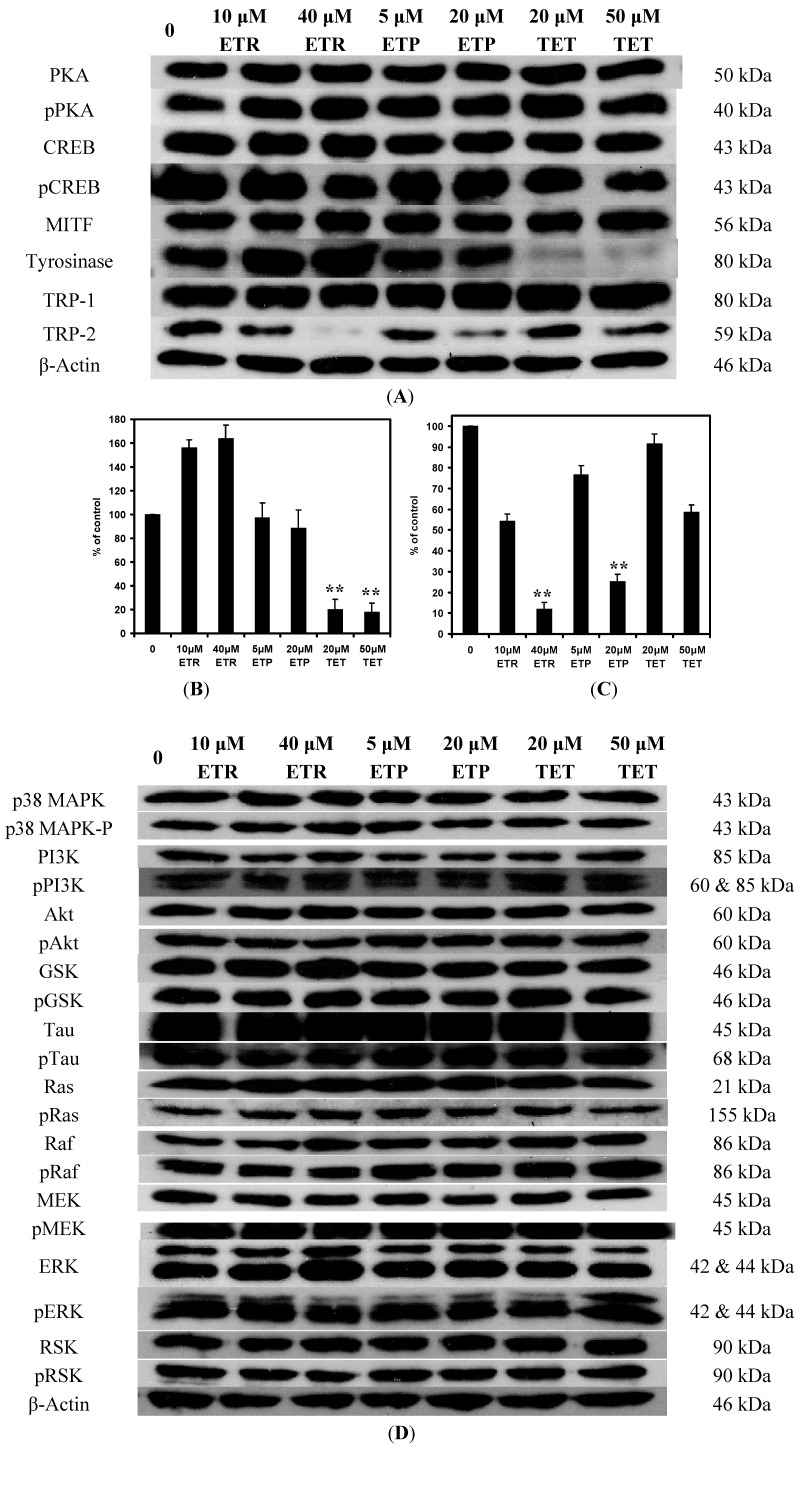
(**A**) Western blot analysis on the effects of 4-ethylresorcinol, 4-ethylphenol and 1-tetradecanol on the protein expression level of PKA pathway. β-actin was used as a control for the system; Graphical presentation of the effects of 4-ethylresorcinol, 4-ethylphenol and 1-tetradecanol on tyrosinase (**B**) and TRP-2 (**C**) protein expression. The densitometry of tyrosinase and TRP-2 protein expression was analyzed by AlphaEaseFC software (Alpha Innotech Corporation, San Leandro, CA, USA) and presented in a graphical expression. The quantified densitometry was expressed as percentage of control. **, *p* < 0.01 when compared to control using one-way Analysis of Variance (ANOVA) followed by Dunnett’s test; (**D**) Western blot analysis on the effects of 4-ethylresorcinol, 4-ethylphenol and 1-tetradecanol on the protein expression level of p38 MAPK pathway, PI3K pathway and ERK pathway. ETR, 4-ethylresorcinol; ETP, 4-ethylphenol; TET, 1-tetradecanol; PKA, protein kinase A; CREB, cAMP response element binding protein; MITF, microphthalmia-associated transcription factor; TRP, tyrosinase-related protein; MAPK, mitogen-activated protein kinase; PI3K, phosphatidylinositol 3-kinase; GSK, glycogen synthase kinase; MEK, MAPK kinase; ERK, extracellular signal-regulated kinase; RSK, ribosomal S6 kinase.

The effects of 4-ethylresorcinol, 4-ethylphenol and 1-tetradecanol on the protein expression level of several signaling pathways were shown in Figure 2. The concentrations of these compounds applied in our experiments were the effective concentrations to induce hypopigmentation in melan-a cells without causing cytotoxicity. Regarding the Western blot analysis on the effects of the chemical compounds on the PKA pathway (Figure 2A), it could be observed that 4-ethylresorcinol and 4-ethylphenol exhibited inhibitory effects on TRP-2 expression and 1-tetradecanol inhibited the expression of tyrosinase at higher concentration. The quantifications of the observations were shown in Figure 2B,C. Other proteins in the same pathway showed no obvious alteration in the presence of the three compounds. As shown in Figure 2D, expressions of the proteins in the p38 MAPK pathway, PI3K pathway and ERK pathway remained unchanged under the effect of the compounds.

### 2.2. Effects of the Chemical Compounds on Gene Expression Level in Melan-a Cells

**Figure 3:**
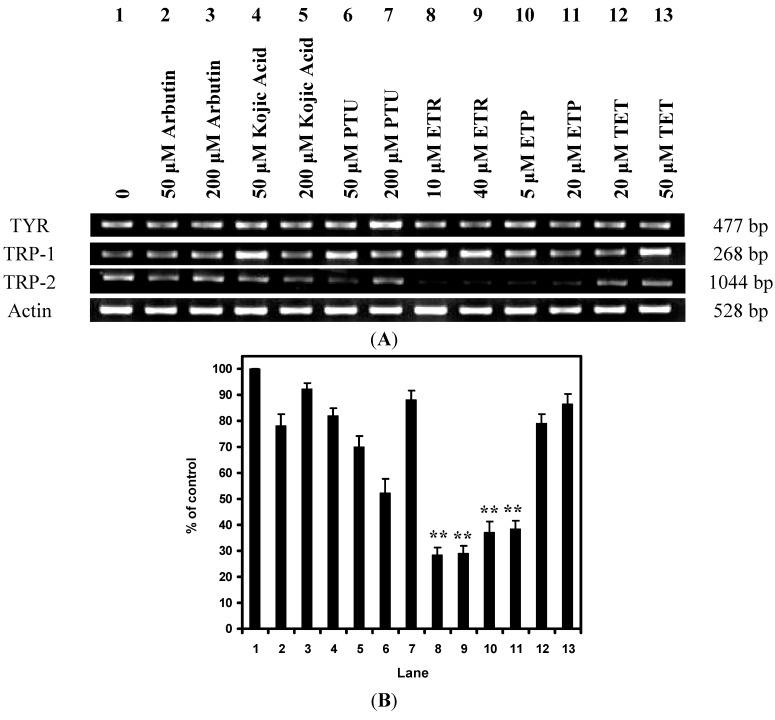
(**A**) Effects of 4-ethylresorcinol, 4-ethylphenol and 1-tetradecanol on the mRNA expression of tyrosinase family genes. Actin was used as a control for the system. Arbutin, kojic acid and PTU were applied for reference; (**B**) Graphical presentation of the effects of 4-ethylresorcinol, 4-ethylphenol and 1-tetradecanol on the mRNA expression of TRP-2. The densitometry of *TRP-2* gene expression was analyzed by AlphaEaseFC software (Alpha Innotech Corporation) and presented in a graphical expression. The quantified densitometry was expressed as percentage of control. **, *p* < 0.01 when compared to control using one-way ANOVA followed by Dunnett’s test. PTU, phenylthiourea; ETR, 4-ethylresorcinol; ETP, 4-ethylphenol; TET, 1-tetradecanol; TYR, tyrosinase; TRP, tyrosinase-related protein.

The effects of 4-ethylresorcinol, 4-ethylphenol and 1-tetradecanol on the mRNA expression levels of tyrosinase, TRP-1 and TRP-2 were shown in Figure 3A. It could be observed that 4-ethylresorcinol and 4-ethylphenol decreased *TRP-2* gene expression at both concentrations. The quantification of the results was shown in Figure 3B. The established depigmenting agents arbutin, kojic acid and phenylthiourea (PTU) were used as controls, which showed no inhibition on the level of the *TRP-2* gene. Other genes of the same protein family, including *tyrosinase* and *TRP-1*, remained unchanged in their expression levels upon incubation with the three compounds and the controls. Moreover, 1-tetradecanol did not alter the expression level of *tyrosinase*, *TRP-1* and *TRP-2* genes.

### 2.3. Effects of the Chemical Compounds on Cyclic Adenosine Monophosphate (cAMP) Level in Melan-a Cells

**Figure 4:**
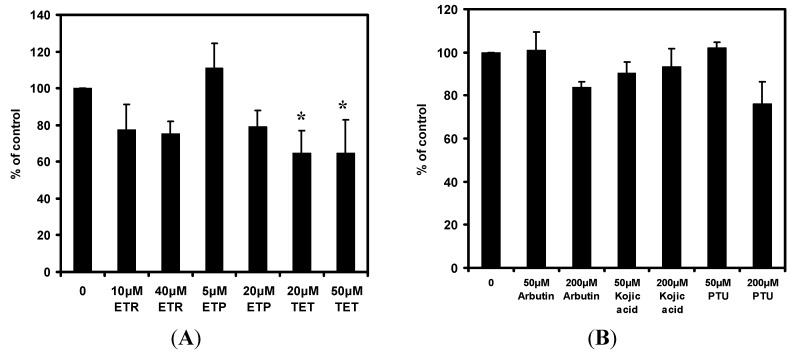
Effects of 4-ethylresorcinol, 4-ethylphenol, 1-tetradecanol (**A**); arbutin, kojic acid and PTU (**B**) on cAMP level in melan-a cells. The extent of cAMP level attenuation was expressed as percentage of control. Each data point presents mean ± SD from three independent experiments. *, *p* < 0.05 when compared to control using one-way ANOVA followed by Dunnett’s test. Arbutin, kojic acid and PTU were applied for reference. ETR, 4-ethylresorcinol; ETP, 4-ethylphenol; TET, 1-tetradecanol; PTU, phenylthiourea.

The effects of 4-ethylresorcinol, 4-ethylphenol and 1-tetradecanol on cAMP level in melan-a cells were shown in Figure 4A. Among the compounds, 1-tetradecanol exhibited the strongest inhibitory effect (nearly 40% inhibition) on cAMP level at both concentrations. This is followed by 4-ethylresorcinol in which the inhibition percentage was about 25% at both concentrations. 4-Ethylphenol had approximately 20% inhibition at high concentration. The common depigmenting agents arbutin, kojic acid and phenylthiourea (PTU) elicited less than 25% inhibition on cAMP level (Figure 4B). Kojic acid slightly augmented cAMP level when dosage increased. PTU produced only about 24% inhibition at high concentration and the concentration of PTU that elicited inhibition on cAMP level was much higher than those of the three chemical compounds. It is clear that the three chemical compounds had stronger attenuating effect on cAMP level than the controls.

### 2.4. Effects of the Chemical Compounds on Protein Kinase A (PKA) Activity in Melan-a Cells

**Figure 5:**
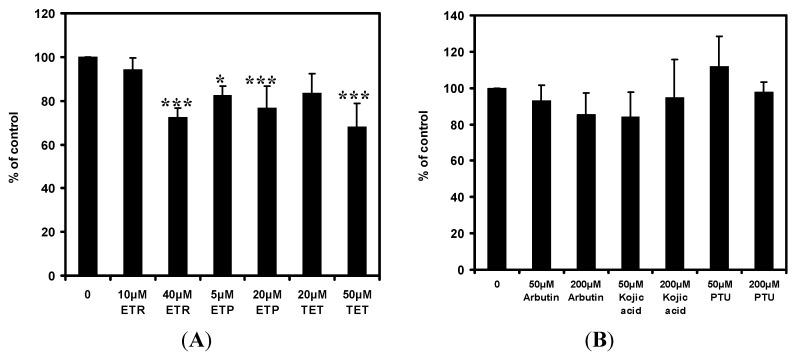
Effects of 4-ethylresorcinol, 4-ethylphenol, 1-tetradecanol (**A**); arbutin, kojic acid and PTU (**B**) on PKA activity in melan-a cells. The extent of PKA activity attenuation was expressed as percentage of control. Each data point presents mean ± SD from three independent experiments. *, *p* < 0.05; ***, *p* < 0.001 when compared to control using one-way ANOVA followed by Dunnett’s test. Arbutin, kojic acid and PTU were applied for reference. ETR, 4-ethylresorcinol; ETP, 4-ethylphenol; TET, 1-tetradecanol; PTU, phenylthiourea.

The effects of 4-ethylresorcinol, 4-ethylphenol and 1-tetradecanol on the activity of PKA in melan-a cells were shown in Figure 5A. All three compounds exhibited different degrees of inhibition on the activity of PKA. They were able to attenuate PKA activity at about 30% and 20% at high and low concentrations respectively. 1-Tetradecanol at high concentration exerted the strongest inhibition on PKA activity among the three compounds, followed by 4-ethylresorcinol and 4-ethylphenol. The common depigmenting agents arbutin, kojic acid and PTU had less than 20% inhibition on PKA activity (Figure 5B). PTU had no marked inhibition while kojic acid showed small activation on PKA activity when its concentration increased. Thus, the inhibitory effects of the depigmenting agents on PKA activity were weaker than the three chemical compounds.

### 2.5. Effects of the Chemical Compounds on Tyrosinase Enzyme Activity

**Figure 6:**
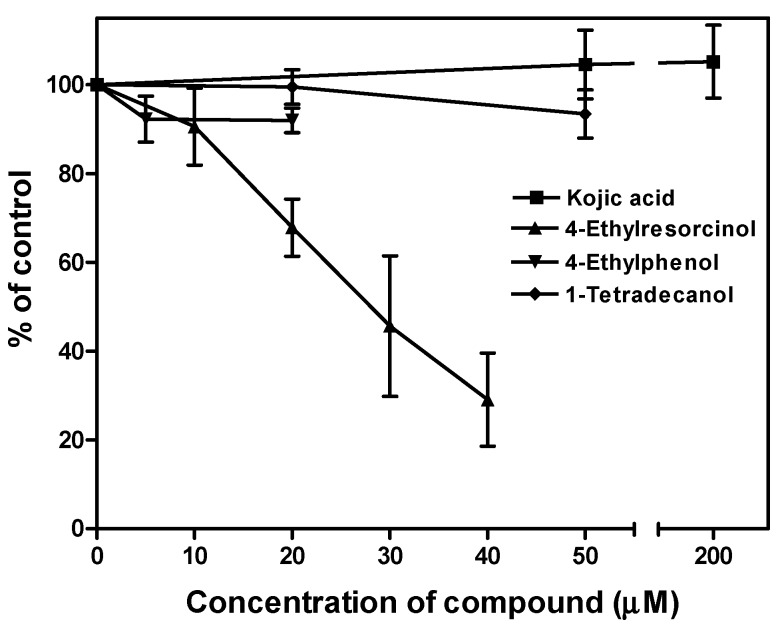
Effects of 4-ethylphenol and 1-tetradecanol on tyrosinase activity of melan-a cells. The extent of enzyme inhibition was expressed as percentage of control. Each data point presents mean ± SD from three independent experiments. 4-Ethylresorcinol and kojic acid were applied for reference.

The inhibitory effects of the three compounds on tyrosinase activity of melan-a cells were depicted in Figure 6. 4-Ethylresorcinol was a control in which its IC_50_ was found to be 21.1 μM in our assay condition. The activity of tyrosinase remained unchanged after the incubation of 4-ethylphenol and 1-tetradecanol.

### 2.6. Effects of the Chemical Compounds on Lipid Peroxidation of Mouse Liver Microsome

**Figure 7:**
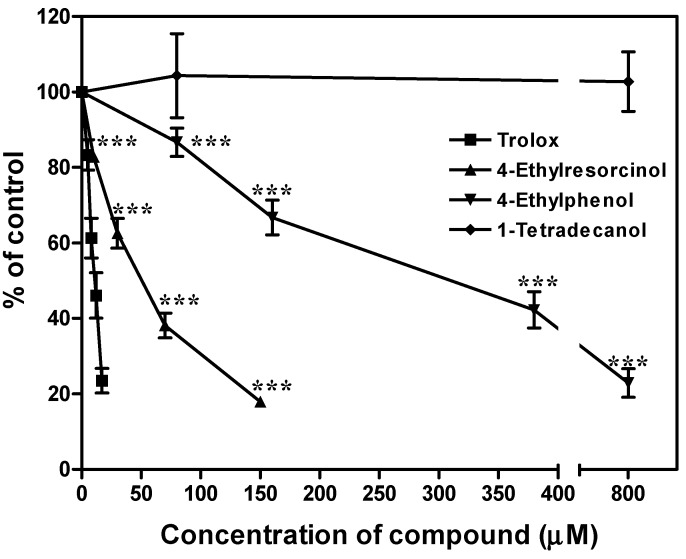
Effects of 4-ethylresorcinol, 4-ethylphenol and 1-tetradecanol on lipid peroxidation inhibition. The extent of lipid peroxidation inhibition was expressed as percentage of control. Each data point presents mean ± SD from three independent experiments. ***, *p* < 0.001 when compared to control using one-way ANOVA followed by Dunnett’s test. Trolox was applied for reference.

The antioxidative ability of the hypopigmentary compounds on 2,2'-azobis (2-amidinopropane) dihydrochloride (AAPH)-induced mouse liver microsome peroxidation was shown in Figure 7. The amount of colored complex decreased dose-dependently under the effect of 4-ethylresorcinol and 4-ethylphenol with IC_50_ values of 38.4 and 234.6 μM respectively. The colored complex amount remained constant in the presence of 1-tetradecanol. Trolox was used as a positive control, and its IC_50_ was found to be 8.6 μM in our assay condition.

## 3. Discussion

Based on the melanin production screening assay in our previous work [26], 4-ethylresorcinol, 4-ethylphenol and 1-tetradecanol were found to possess hypopigmentary effects. In the present study, we aimed to elucidate the underlying hypopigmentary mechanisms of these compounds. All three compounds attenuated the protein expression levels of tyrosinase family proteins (Figure 2A). Reduction in protein expression may influence the cascade reaction of melanogenesis and lead to depigmentation. The inhibitory effects of 4-ethylresorcinol and 4-ethylphenol on TRP-2 expression may suppress the tautomerization of dopachrome to DHICA [10], and the inhibition of 1-tetradecanol on tyrosinase expression may restrain the conversion of tyrosine to DOPA [4]. Inhibitory effects of effective compounds on melanogenic protein expressions may be due to the abilities of the compounds to affect translation or induce degradation of the proteins [27]. These possible factors contribute to the hypopigmentary effects of the compounds, since TRP-2 and tyrosinase control critical steps of melanin synthesis which takes place in the melanosomes of melanocytes. Further evidence is needed to delineate the precise mechanism of action of the compounds towards tyrosinase and TRP-2 expression.

4-Ethylresorcinol, 4-ethylphenol and 1-tetradecanol have a hydroxyl group in common, they all exert inhibitory effects on tyrosinase family proteins. 4-Ethylresorcinol and 4-ethylphenol have high similarity within their chemical structures, which differ in one hydroxyl group only, and their high similarities may be the reason that they exerted similar effect on TRP-2. Although tyrosinase, TRP-1 and TRP-2 belong to the same protein family, they do have variations in their protein structures [28,29,30], and inhibition on one of them does not mean all of them can be inhibited. This may elucidate the different inhibiting targets of the compounds. Another interesting finding from our present study is that the protein expression level of tyrosinase was increased under the effect of 4-ethylresorcinol (Figure 2B). Since 4-ethylresorcinol inhibited TRP-2 expression (Figure 2C) and attenuated melanin synthesis, its accentuating role on tyrosinase protein expression remains to be determined. Moreover, the three compounds had no obvious influence on the protein expression levels of p38 MAPK, PI3K and ERK pathways (Figure 2D). This provided opposite arguments for the compounds which verified that their depigmenting effects did not affect p38 MAPK, PI3K and ERK pathways.

Since the compounds exerted effects on the expression of tyrosinase family proteins, their effects on the expression of tyrosinase family genes were examined. 4-Ethylresorcinol and 4-ethylphenol were found to attenuate the mRNA expression of TRP-2 (Figure 3A). When transcription of the *TRP-2* gene was inhibited and the mRNA level was alleviated under the effect of the two compounds, it may modulate the TRP-2 protein production dogma by terminating subsequent translation and translational modification of TRP-2 protein. This inference was supported by the results from Western blot analysis which demonstrated the inhibitory effect of 4-ethylresorcinol and 4-ethylphenol on TRP-2 protein expression. Therefore, the underlying hypopigmentary mechanism of the two compounds turned out to be the inhibition at the transcriptional level of TRP-2.

The effects of 4-ethylresorcinol and 4-ethylphenol were compared to those of the known depigmenting agents including arbutin, kojic acid and PTU. The two compounds showed significant attenuating effect on *TRP-2* gene expression while the controls did not. Moreover, the two compounds did not affect the gene expression of *tyrosinase* and *TRP-1* (Figure 3A). The same result occurred on the protein expression of tyrosinase and TRP-1 (Figure 2A). Among the tyrosinase family genes, only *TRP-2* gene expression was inhibited; *tyrosinase* and *TRP-1* gene expression remained unaffected by all the test compounds (Figure 3A). 4-Ethylresorcinol and 4-ethylphenol acted on TRP-2 effectively at both gene and protein levels. They attenuated *TRP-2* gene expression at both concentrations, but down-regulated TRP-2 protein expression only at higher concentration, thus their effect on TRP-2 protein expression appeared to be dose-dependent. Furthermore, 1-tetradecanol, which has a chemical structure different from the other two compounds, did not affect the mRNA level of the tyrosinase family gene (Figure 3A), though it alleviated tyrosinase protein expression at higher concentration (Figure 2A). 1-Tetradecanol was able to attenuate tyrosinase protein expression, but it could not affect the transcription of *tyrosinase* gene, so the attenuation in tyrosinase protein expression was not due to attenuation in *tyrosinase* gene expression. It appeared that the hypopigmentary effect of 1-tetradecanol was limited to the translational level of tyrosinase.

4-Ethylresorcinol, 4-ethylphenol and 1-tetradecanol were found to attenuate cAMP level, with 1-tetradecanol being the most potent, followed by 4-ethylresorcinol and 4-ethylphenol (Figure 4A). Moreover, the compounds exhibited stronger effects in decreasing cAMP amount than the common depigmenting agents did (Figure 4B). Although PTU could achieve similar effects with the compounds, its concentration was much higher than the tested compounds. On the other hand, PKA is a critical enzyme responsible for the regulation of numerous cellular pathways within cells [31]. It affects the activity of a number of signaling proteins along the melanogenic pathway. Our results showed that 4-ethylresorcinol, 4-ethylphenol and 1-tetradecanol were able to inhibit the PKA activity (Figure 5A). Among the three compounds, 1-tetradecanol had the strongest inhibition at high concentration, followed by 4-ethylresorcinol and 4-ethylphenol. The order of inhibitory ability was similar to that of the cAMP assay. The inhibition percentages of the compounds on PKA activity were higher than the common depigmenting agents (Figure 5B).

The compounds were able to attenuate PKA activity after decreasing cAMP level. This is reasonable as PKA activation depends on cAMP. Our results confirmed that all three compounds inhibited PKA activity in a cAMP-dependent manner and their effects in attenuating cAMP level and PKA activity were stronger than that of the known depigmenting agents. Although the compounds attenuated cAMP amount and PKA activity, the protein expression of the subsequent signaling proteins in the pathway, CREB and MITF, remained unchanged under the influence of the compounds (Figure 2A). As a result, the effect of the three compounds on cAMP and PKA activity may contribute to hypopigmentation indirectly through other pathways as cAMP is a common second messenger of many signaling cascades. Our experimental findings corroborated that the compounds acted on cAMP and PKA, the compounds also acted on tyrosinase and TRP-2 which are downstream proteins that participate in the pathway cascade initiated by PKA [16], thus it is believed that the compounds commonly affected the PKA pathway.

Tyrosinase is the rate-limiting enzyme of melanogenesis. The enzyme assay of tyrosinase is convenient and efficient, and is commonly used as a screening tool in pigmentation research. Many compounds have been found to inhibit tyrosinase activity [32,33,34,35,36,37]. The competitive inhibition of 4-ethylresorcinol towards tyrosinase was reported previously [35]. Our tyrosinase assay elucidates that known tyrosinase inhibitors (such as kojic acid) might take time to attain their effects in clinical conditions and their degree of persistency could be revealed.

The discrepancies between 4-ethylresorcinol and 4-ethylphenol in terms of their inhibitory actions on tyrosinase may be due to different numbers and positions of functional groups on the benzene ring. 4-Ethylresorcinol possesses two hydroxyl groups at para (*p*) and ortho (*o*) positions, respectively. The compound is electron-rich, as electron delocalization occurs between lone pair electrons of the phenoxyl oxygen and π electrons in the benzene ring. This may allow the compound to interact with the copper ion active site and interfere with the activity of tyrosinase [38]. 4-Ethylphenol lacks a hydroxyl group at position *o* of the benzene ring when compared to 4-ethylresorcinol. Its electron density was lower than 4-ethylresorcinol during electron delocalization, so it could barely interact with the active site of tyrosinase, and thus inhibition was not possible. Hence, the *o* position hydroxyl group is essential for the inhibition of tyrosinase activity. Since the hydroxyl group has electron-donating properties, more hydroxyl groups in 4-ethylresorcinol compared with 4-ethylphenol, resulted in greater inhibitory effects; this showed the significance of the number of hydroxyl groups in tyrosinase inhibition [39]. 1-Tetradecanol does not have a benzene ring structure, and though it contains a hydroxyl group, electron delocalization cannot occur without π electrons. It was not electron-dense enough to interact with tyrosinase; this may explain its ineffectiveness towards tyrosinase inhibition.

Many previous studies only tested the effect of chemicals on mushroom tyrosinase inhibition and chemicals with positive effect are regarded as potential whitening agents. In fact, results from mushroom tyrosinase screening are preliminary which requires confirmation by cell-based methods, as some hypopigmentary compounds may not be tyrosinase inhibitors, for example 4-ethylphenol and 1-tetradecanol (Figure 6). The advantage of our melan-a cell screening system over the mushroom tyrosinase screening method is that melan-a mimics a high hyperpigmentary condition in normal skin devoid of hormonal stimulation; thus the screened chemicals with positive effects have ensured ability to attenuate melanin production for further* in vivo* study.

Depigmenting agents with antioxidative abilities are also particularly ideal because oxidation can bring about an increase in the degree of hyperpigmentation. When there is cumulative UV exposure, free radicals are produced within cells owing to induction of lipid peroxidation of skin cell membrane [36]. The situation may accelerate melanogenesis, as melanin can be synthesized by autooxidation of metabolites independent of tyrosinase family enzymes. Searching for depigmenting agents that play a role as an antioxidant at the same time helps to scavenge any free radicals present in skin cells. This would thereby attenuate the metabolite autooxidation which initiates subsequent melanin formation, and terminates free radical oxidation of cell membranes that leads to post-inflammatory hyperpigmentation and cell aging [40]. The antioxidant ability of the compounds was assessed by the mouse liver microsome lipid peroxidation inhibition assay. In this assay, mouse liver microsome acting as a lipid source is oxidized by the oxidant AAPH. Once oxidation takes place, lipid peroxides and malonyldialdehyde (MDA) are produced as a result of lipid peroxidation [41,42].

4-Ethylresorcinol and 4-ethylphenol attenuated the production of MDA complex in a dose-dependent manner (Figure 7), suggesting that 4-ethylresorcinol and 4-ethylphenol were antioxidants as they were able to scavenge AAPH-induced radicals and terminate chain reaction propagation of lipid peroxidation. Among the two compounds, 4-ethylresorcinol was a better radical scavenger and chain terminator than 4-ethylphenol. Variation in antioxidative effect of the two compounds may be attributed to the number and position of the functional groups [43]. 4-Ethylresorcinol was a stronger antioxidant as it has one more hydroxyl group than 4-ethylphenol; it was able to form phenoxyl radical more readily than 4-ethylphenol due to stronger resonance. This allowed it to interact with free radicals and stop oxidation propagation [44]. For instance, 4-ethylresorcinol and 4-ethylphenol had IC_50_ values higher than that of the positive control trolox, an effective antioxidant. 1-Tetradecanol lacked antioxidant activity, perhaps due to its linear structure, which was unable to form phenoxyl radicals to interact with the free radicals. As the oxidation product in this assay system could hardly be detected at minute quantities, higher concentrations of lipid sources and oxidants had to be applied during incubations; thus higher concentrations of compounds were required to reach the threshold of antioxidative effect. Therefore, the compounds that possessed antioxidative effects in this assay might be effective at low concentrations when the amount of lipid source and oxidant are lower in the cellular condition.

The results of our present mechanistic study indicated that 4-ethylresorcinol is more effective in inhibiting melanin synthesis when compared to the other two compounds [26]. 4-Ethylresorcinol was able to attenuate mRNA and protein expression of TRP-2 (Figure 2A and Figure 3A) and inhibit tyrosinase activity [35], which probably overrode the effect of elevated protein expression of tyrosinase (Figure 2A). It also possessed antioxidative effects by inhibiting lipid peroxidation (Figure 7). These underlying mechanisms in different aspects render 4-ethylresorcinol with good anti-melanogenic advantages over 4-ethylphenol and 1-tetradecanol; 4-ethylphenol only attenuated mRNA and protein expression of TRP-2 (Figure 2A and Figure 3A), and possessed antioxidative effect (Figure 7), while 1-tetradecanol was only effective at the post-transcriptional level by attenuating protein expression of tyrosinase (Figure 2A) without inhibiting tyrosinase catalytic activity (Figure 6).

## 4. Experimental Section

### 4.1. Materials

Apparatus for Western blot analysis was purchased from Bio-Rad (Hercules, CA, USA), except the film exposure cassette was from Amersham Biosciences Corporation (Piscataway, NJ, USA). Primary antibodies were purchased from Santa Cruz Biotechnology, Inc. (Santa Cruz, CA, USA), Invitrogen Corporation (Carlsbad, CA, USA) and Cell Signaling Technology, Inc. (Danvers, MA, USA). Secondary antibodies were purchased from Santa Cruz Biotechnology, Inc. (Santa Cruz). Trizol was purchased from Invitrogen Corporation (Carlsbad). RT system was purchased from Promega (Madison, WI, USA). PCR machine was the product of Takara Bio Inc. (Otsu, Shiga, Japan). NanoDrop 2000c spectrophotometer for nucleic acid measurement was purchased from Thermo Scientific (Wilmington, DE, USA). The cAMP EIA kit was purchased from Cayman Chemical Company (Ann Arbor, MI, USA). Bio-Rad protein dye was purchased from Bio-Rad (Hercules). The PKA kinase activity kit was purchased from Enzo Life Sciences Inc. (Farmingdale, NY, USA). DOPA was purchased from Sigma–Aldrich (St. Louis, MO, USA). Other chemicals used in the study were purchased from Sigma–Aldrich (St. Louis) and USB (Cleveland, OH, USA).

### 4.2. Cell Culture

Melan-a is a copiously pigmented, immortalized non-tumorigenic mouse melanocyte cell line established from normal epidermal melanoblasts of embryos of inbred C57BL mice. Melan-a cells were grown in RPMI1640 medium supplemented with 10% FBS, 100 μg/mL streptomycin, 50 U/mL penicillin and 200 nM tetradecanoyl phorbol acetate (TPA) and incubated at 37 °C in a 5% CO_2_ and 95% air humidified atmosphere. When cells were incubated with the chemical compounds, 20 nM TPA was used in the incubation medium for maintenance of cell growth [26].

### 4.3. Western Blot Analysis

#### 4.3.1. Preparation of Cell Lysates

After five days of continuing incubation with chemical compounds, the culture medium was discarded from the 12-well plate, the melan-a cells were washed twice with phosphate buffered saline (PBS). Then 100 μL trypsin was added to each well and the plate was incubated at 37 °C for 10 min. Trypsinization was stopped by adding 100 μL of medium to each well, and the cells were scraped and collected in microtubes. The microtubes were centrifuged at 2000× *g* for 3 min, and the cell pellets were retained for each sample. All pellets were resuspended with lysis buffer containing 50 mM Tris base (pH 7.5), 0.1 M sodium chloride (NaCl), 5 mM EDTA, 67 mM sodium pyrophosphate, 1.5% Triton X-100, 0.2 mM sodium orthovanadate, 0.2 mM phenylmethylsulfonyl fluoride, 1 mM dithiothreitol, 4 μM leupeptin and 4 μg/mL aprotinin. The cells were lysed on ice for 30 min with repeated pipetting action and the lysates were centrifuged at 12,000× *g* for 15 min at 4 °C. The supernatant was transferred to another microtube, followed by adding loading buffer and the mixture was boiled at 100 °C for 15 min with gentle shaking [45,46].

#### 4.3.2. Protein Assay

The current protein assay protocol was a summarized modification of the Bio-Rad protein assay. Briefly, the protein dye was diluted to its working concentration and was filtered. Then 10 μL diluted samples, together with various concentrations of bovine serum albumin standards (0, 0.025, 0.05, 0.1, 0.2, 0.4, 0.6 and 0.8 mg/mL) were transferred to a 96-well plate. After that, 200 μL diluted protein dye was added to each well and absorbance was read at 595 nm.

#### 4.3.3. Western Blot

The cell lysates were loaded (70–80 μg/lane) and separated by sodium dodecyl sulfate polyacrylamide gel electrophoresis (SDS-PAGE) with 4% stacking gel and 10% separating gel for 90 min. The gels, together with polyvinylidene fluoride (PVDF) membranes (Millipore Corporation, Billerica, MA, USA) which were presoaked in methanol, then in transfer buffer (25 mM Tris pH 8.3, 192 mM glycine and 20% methanol), were placed in a semi-dry transfer system (Bio-Rad, Hercules) and transferred for 70 min. The membranes were then blocked for 1 h by 5% milk in Tris-buffered saline-Tween (TTBS) containing 20 mM Tris (pH 7.5), 0.8% NaCl and 0.1% Tween 20. After that, the membranes were incubated overnight with primary antibodies at 4 °C with constant shaking. The next day, the membranes were washed with TTBS and incubated with corresponding secondary antibodies for 1 h at room temperature with constant shaking, followed by another wash. Chemiluminescent horseradish peroxidase (HRP) substrate (Millipore Corporation, Billerica, MA, USA) was used for film development to visualize blotted images [45,47].

### 4.4. RT-PCR

Total RNA was extracted from cells using Trizol solution according to the manufacturer’s instructions (Invitrogen Corporation, Carlsbad). RNA concentrations were measured by NanoDrop 2000c spectrophotometer (Thermo Scientific). RT and PCR were performed by using 1 μg of total RNA as per the manufacturer’s protocol (Promega, Madison, WI, USA). The following oligonucleotide primers were applied in PCR: tyrosinase upstream 5'-GGC CAG CTT TCA GGC AGA GGT-3'; downstream 5'-TGG TGC TTC ATG GGC AAA ATC-3'; TRP-1 upstream 5'-GCT GCA GGA GCC TTC TTT CTC-3'; downstream 5'-AAG ACG CTG CAC TGC TGG TCT-3'; TRP-2 upstream 5'-GGA TGA CCG TGA GCA ATG GCC-3'; downstream 5'-CGG TTG TGA CCA ATG GGT GCC-3'; Actin upstream 5'-TGG AAT CCT GTG GCA TCC ATG AAA C-3'; downstream 5'-TAA AAC GCA GCT CAG TAA CAG TCC G-3'. The PCR reaction was cycled 25 times: 94 °C, 30 s; 56 °C, 30 s; 72 °C, 45 s. PCR products were electrophoresed in 1% agarose gel, and the bands were visualized by a UV illuminator [48,49,50].

### 4.5. cAMP Assay

cAMP assay was performed according to the manufacturer’s instructions (Cayman Chemical Company). Briefly, the cells were lysed on ice for 30 min with repeated pipetting action, and the lysates were centrifuged at 2000× *g* for 5 min at 4 °C. The supernatant was transferred to another microtube and diluted with EIA buffer in a 1:2 ratio. The cAMP concentrations of the samples were determined by the equation obtained from the calibration curve according to the protocol, and the results were expressed as percentage of control.

### 4.6. The PKA Activity Assay

The PKA activity assay was performed according to the manufacturer’s instructions (Enzo Life Sciences Inc., Farmingdale, NY, USA). Briefly, the cells were lysed on ice for 30 min with repeated pipetting action, and the lysates were centrifuged at 12,000× *g* for 15 min at 4 °C. The supernatant was transferred to another microtube. The average absorbance of blank was subtracted from the average absorbance of samples and controls. The corrected sample absorbance was divided by the corrected control absorbance at 450 nm and the results were expressed as the percentage of control.

### 4.7. Tyrosinase Inhibition Assay

The cell lysates, prepared from the lysis buffer as mentioned in Western blot analysis, were frozen and thawed for a few times and were then centrifuged at 12,000× *g* for 15 min at 4 °C. After that, 90 μL supernatant and 10 μL DOPA were added to each well of a 96-well plate. The plate was wrapped with aluminum foil to avoid light exposure and was incubated at 37 °C. Three days later, the plate was transferred to a microplate spectrophotometer (Sunrise, Tecan Group Ltd., Mannedorf, Switzerland), the absorbance was read at the wavelength of 475 nm [46]. The average absorbance of blank was subtracted from the average absorbance of samples and controls. The corrected sample absorbance was divided by the corrected control absorbance at 475 nm and the results were expressed as the percentage of control.

### 4.8. Antioxidative Activity Assay

#### 4.8.1. Preparation of Mouse Liver Microsomes

BALB/c mice were sacrificed by decapitation. Livers were excised and rinsed twice with 0.9% saline at 4 °C. All subsequent preparative procedures were carried out at 4 °C unless otherwise specified. Livers were then homogenized in 0.15 M potassium chloride (KCl). The amount of KCl added to liver was 3 mL/g. The homogenate was centrifuged at 10,000× *g* for 20 min at 4 °C. The supernatant was retained and centrifuged at 100,000× *g* for 1 h at 4 °C. The supernatant was discarded. The pellet was resuspended with KCl. The stock was stored at −20 °C after determination of protein concentration [51].

#### 4.8.2. Lipid Peroxidation Inhibition Assay

Lipid peroxidation of mouse liver microsomes was produced by AAPH oxidation. The reaction mixture had a final volume of 400 μL containing PBS (pH 7.4), 10 mg/mL mouse liver microsome, 0.5 mM EDTA, chemical compound and 10 mM AAPH. The mixtures were incubated at 37 °C for 1 h with constant stirring. After incubation, 200 μL 20% trichloroacetic acid and 200 μL 0.8% thiobarbituric acid were added to each sample. The mixtures were heated for 20 min at 99 °C. They were then centrifuged at 3000× *g* for 5 min and 200 μL supernatant was transferred to a 96-well plate for measurement at a wavelength of 532 nm [52]. Net absorbance was calculated by subtraction of readings between samples and blanks without AAPH. The net sample absorbance was divided by the net control absorbance at 532 nm and the degree of lipid peroxidation for each sample was expressed as the percentage of control.

### 4.9. Statistical Analysis

Data were presented as mean ± SD. Comparison between treatment groups were carried out using one-way ANOVA followed by *post hoc* Dunnett’s test. Statistical analysis was conducted on a GraphPad Prism 4 computer program (GraphPad Software Inc., San Diego, CA, USA). Differences were considered significant when *p* < 0.05 and denoted as *, *p* < 0.05; **, *p* < 0.01; ***, *p* < 0.001.

## 5. Conclusions

Taking all the results into consideration, it can be concluded that the hypopigmentary actions of the effective compounds mainly focus on regulating downstream proteins, tyrosinase and TRP-2, in the PKA pathway. The present study provides a better understanding of the melanogenic mechanism of a number of chemical compounds, and paves a way for further development of effective hypopigmentary agents into cosmetic products with skin whitening properties on a scientific basis.

## References

[1] Im S., Moro O., Peng F., Medrano E.E., Cornelius J., Babcock G., Nordlund J.J., Abdel-Malek Z.A. (1998). Activation of the cyclic AMP pathway by α-melanotropin mediates the response of human melanocytes to ultraviolet B radiation. Cancer Res..

[2] Bertolotto C., Abbe P., Hemesath T.J., Bille K., Fisher D.E., Ortonne J.P., Ballotti R. (1998). Microphthalmia gene product as a signal transducer in cAMP-induced differentiation of melanocytes. J. Cell Biol..

[3] Yasumoto K., Yokoyama K., Takahashi K., Tomita Y., Shibahara S. (1997). Functional analysis of microphthalmia-associated transcription factor in pigment cell-specific transcription of the human tyrosinase family genes. J. Biol. Chem..

[4] Hearing V.J., Tsukamoto K. (1991). Enzymatic control of pigmentation in mammals. FASEB J..

[5] Sanchez-Ferrer A., Rodriguez-Lopez J.N., Garcia-Canovas F., Garcia-Carmona F. (1995). Tyrosinase: A comprehensive review of its mechanism. Biochim. Biophys. Acta.

[6] Korner A., Pawelek J. (1982). Mammalian tyrosinase catalyzes three reactions in the biosynthesis of melanin. Science.

[7] Jimenez-Cervantes C., Solano F., Kobayashi T., Urabe K., Hearing V.J., Lozano J.A., Garcia-Borron J.C. (1994). A new enzymatic function in the melanogenic pathway. The 5,6-dihydroxyindole-2-carboxylic acid oxidase activity of tyrosinase-related protein-1 (TRP1). J. Biol. Chem..

[8] Kobayashi T., Urabe K., Winder A., Jimenez-Cervantes C., Imokawa G., Brewington T., Solano F., Garcia-Borron J.C., Hearing V.J. (1994). Tyrosinase related protein 1 (TRP1) functions as a DHICA oxidase in melanin biosynthesis. EMBO J..

[9] Kobayashi T., Urabe K., Winder A., Tsukamoto K., Brewington T., Imokawa G., Potterf B., Hearing V.J. (1994). DHICA oxidase activity of TRP1 and interactions with other melanogenic enzymes. Pigment. Cell Res..

[10] Tsukamoto K., Jackson I.J., Urabe K., Montague P.M., Hearing V.J. (1992). A second tyrosinase-related protein, TRP-2, is a melanogenic enzyme termed DOPAchrome tautomerase. EMBO J..

[11] Yokoyama K., Suzuki H., Yasumoto K., Tomita Y., Shibahara S. (1994). Molecular cloning and functional analysis of a cDNA coding for human DOPAchrome tautomerase/tyrosinase-related protein-2. Biochim. Biophys. Acta.

[12] Saha B., Singh S.K., Sarkar C., Bera R., Ratha J., Tobin D.J., Bhadra R. (2006). Activation of the Mitf promoter by lipid-stimulated activation of p38-stress signalling to CREB. Pigment. Cell Res..

[13] Singh S.K., Sarkar C., Mallick S., Saha B., Bera R., Bhadra R. (2005). Human placental lipid induces melanogenesis through p38 MAPK in B16F10 mouse melanoma. Pigment. Cell Res..

[14] Busca R., Bertolotto C., Ortonne J.P., Ballotti R. (1996). Inhibition of the phosphatidylinositol 3-kinase/p70(S6)-kinase pathway induces B16 melanoma cell differentiation. J. Biol. Chem..

[15] Cantley L.C. (2002). The phosphoinositide 3-kinase pathway. Science.

[16] Khaled M., Larribere L., Bille K., Aberdam E., Ortonne J.P., Ballotti R., Bertolotto C. (2002). Glycogen synthase kinase 3β is activated by cAMP and plays an active role in the regulation of melanogenesis. J. Biol. Chem..

[17] Takeda K., Takemoto C., Kobayashi I., Watanabe A., Nobukuni Y., Fisher D.E., Tachibana M. (2000). Ser298 of MITF, a mutation site in Waardenburg syndrome type 2, is a phosphorylation site with functional significance. Hum. Mol. Genet..

[18] Gundersen G.G., Cook T.A. (1999). Microtubules and signal transduction. Curr. Opin. Cell Biol..

[19] Busca R., Abbe P., Mantoux F., Aberdam E., Peyssonnaux C., Eychene A., Ortonne J.P., Ballotti R. (2000). Ras mediates the cAMP-dependent activation of extracellular signal-regulated kinases (ERKs) in melanocytes. EMBO J..

[20] Englaro W., Rezzonico R., Durand-Clement M., Lallemand D., Ortonne J.P., Ballotti R. (1995). Mitogen-activated protein kinase pathway and AP-1 are activated during cAMP-induced melanogenesis in B-16 melanoma cells. J. Biol. Chem..

[21] Englaro W., Bertolotto C., Busca R., Brunet A., Pages G., Ortonne J.P., Ballotti R. (1998). Inhibition of the mitogen-activated protein kinase pathway triggers B16 melanoma cell differentiation. J. Biol. Chem..

[22] Hemesath T.J., Price E.R., Takemoto C., Badalian T., Fisher D.E. (1998). MAP kinase links the transcription factor Microphthalmia to c-Kit signalling in melanocytes. Nature.

[23] Kim Y.J., No J.K., Lee J.H., Chung H.Y. (2005). 4,4'-Dihydroxybiphenyl as a new potent tyrosinase inhibitor. Biol. Pharm. Bull..

[24] Wu M., Hemesath T.J., Takemoto C.M., Horstmann M.A., Wells A.G., Price E.R., Fisher D.Z., Fisher D.E. (2000). c-Kit triggers dual phosphorylations, which couple activation and degradation of the essential melanocyte factor Mi. Genes Dev..

[25] Xu W., Gong L., Haddad M.M., Bischof O., Campisi J., Yeh E.T., Medrano E.E. (2000). Regulation of microphthalmia-associated transcription factor MITF protein levels by association with the ubiquitin-conjugating enzyme hUBC9. Exp. Cell Res..

[26] Lam R.Y., Lin Z.X., Sviderskaya E., Cheng C.H. (2010). Application of a combined sulphorhodamine B and melanin assay to the evaluation of Chinese medicines and their constituent compounds for hyperpigmentation treatment. J. Ethnopharmacol..

[27] Kageyama A., Oka M., Okada T., Nakamura S., Ueyama T., Saito N., Hearing V.J., Ichihashi M., Nishigori C. (2004). Down-regulation of melanogenesis by phospholipase D2 through ubiquitin proteasome-mediated degradation of tyrosinase. J. Biol. Chem..

[28] Bertolotto C., Busca R., Abbe P., Bille K., Aberdam E., Ortonne J.P., Ballotti R. (1998). Different cis-acting elements are involved in the regulation of TRP1 and TRP2 promoter activities by cyclic AMP: Pivotal role of M boxes (GTCATGTGCT) and of microphthalmia. Mol. Cell Biol..

[29] Jackson I.J., Chambers D.M., Budd P.S., Johnson R. (1991). The tyrosinase-related protein-1 gene has a structure and promoter sequence very different from tyrosinase. Nucleic Acids Res..

[30] Sturm R.A., O’Sullivan B.J., Box N.F., Smith A.G., Smit S.E., Puttick E.R., Parsons P.G., Dunn I.S. (1995). Chromosomal structure of the human TYRP1 and TYRP2 loci and comparison of the tyrosinase-related protein gene family. Genomics.

[31] Kim S., Lee J., Jung E., Lee J., Huh S., Hwang H., Kim Y., Park D. (2009). 6-Benzylaminopurine stimulates melanogenesis via cAMP-independent activation of protein kinase A. Arch. Dermatol. Res..

[32] Khatib S., Nerya O., Musa R., Shmuel M., Tamir S., Vaya J. (2005). Chalcones as potent tyrosinase inhibitors: The importance of a 2,4-substituted resorcinol moiety. Bioorg. Med. Chem..

[33] Kim D.S., Kim S.Y., Park S.H., Choi Y.G., Kwon S.B., Kim M.K., Na J.I., Youn S.W., Park K.C. (2005). Inhibitory effects of 4-*n*-butylresorcinol on tyrosinase activity and melanin synthesis. Biol. Pharm. Bull..

[34] Kim Y.J. (2007). Antimelanogenic and antioxidant properties of gallic acid. Biol. Pharm. Bull..

[35] Shimizu K., Kondo R., Sakai K. (2000). Inhibition of tyrosinase by flavonoids, stilbenes and related 4-substituted resorcinols: Structure-activity investigations. Planta Med..

[36] Shimizu K., Kondo R., Sakai K., Takeda N., Nagahata T., Oniki T. (2001). Novel vitamin E derivative with 4-substituted resorcinol moiety has both antioxidant and tyrosinase inhibitory properties. Lipids.

[37] Shimizu K., Yasutake S., Kondo R. (2003). A new stilbene with tyrosinase inhibitory activity from *Chlorophora excelsa*. Chem. Pharm. Bull..

[38] Medvedev A.E., Ivanov A.S., Kamyshanskaya N.S., Kirkel A.Z., Moskvitina T.A., Gorkin V.Z., Li N.Y., Marshakov V.Y. (1995). Interaction of indole derivatives with monoamine oxidase A and B. Studies on the structure-inhibitory activity relationship. Biochem. Mol. Biol. Int..

[39] Stupans I., Ryan A.J. (1984). *In vitro* inhibition of 3-methylcholanthrene-induced rat hepatic aryl hydrocarbon hydroxylase by 8-acyl-7-hydroxycoumarins. Structure-activity relationships and metabolite profiles. Biochem. Pharmacol..

[40] Lam R.Y., Woo A.Y., Leung P.S., Cheng C.H. (2007). Antioxidant actions of phenolic compounds found in dietary plants on low-density lipoprotein and erythrocytes* in vitro*. J. Am. Coll. Nutr..

[41] Gutteridge J.M. (1975). The use of standards for melonyldialdehyde. Anal. Biochem..

[42] Messina M.J. (1991). Oxidative stress status and cancer: Methodology applicable for human studies. Free Radic. Biol. Med..

[43] Cao H., Pan X., Li C., Zhou C., Deng F., Li T. (2003). Density functional theory calculations for resveratrol. Bioorg. Med. Chem. Lett..

[44] Nakao K., Shimizu R., Kubota H., Yasuhara M., Hashimura Y., Suzuki T., Fujita T., Ohmizu H. (1998). Quantitative structure-activity analyses of novel hydroxyphenylurea derivatives as antioxidants. Bioorg. Med. Chem..

[45] Lin Y.P., Hsu F.L., Chen C.S., Chern J.W., Lee M.H. (2007). Constituents from the Formosan apple reduce tyrosinase activity in human epidermal melanocytes. Phytochemistry.

[46] Park S.H., Kim D.S., Kim W.G., Ryoo I.J., Lee D.H., Huh C.H., Youn S.W., Yoo I.D., Park K.C. (2004). Terrein: A new melanogenesis inhibitor and its mechanism. Cell Mol. Life Sci..

[47] Wu M., Stockley P.G., Martin W.J. (2002). An improved Western blotting technique effectively reduces background. Electrophoresis.

[48] Choi Y.G., Bae E.J., Kim D.S., Park S.H., Kwon S.B., Na J.I., Park K.C. (2006). Differential regulation of melanosomal proteins after hinokitiol treatment. J. Dermatol. Sci..

[49] Jung G.D., Yang J.Y., Song E.S., Par J.W. (2001). Stimulation of melanogenesis by glycyrrhizin in B16 melanoma cells. Exp. Mol. Med..

[50] Koo J.H., Kim H.T., Yoon H.Y., Kwon K.B., Choi I.W., Jung S.H., Kim H.U., Park B.H., Park J.W. (2008). Effect of xanthohumol on melanogenesis in B16 melanoma cells. Exp. Mol. Med..

[51] Dasgupta T., Fabry M.E., Kaul D.K. (2010). Antisickling property of fetal hemoglobin enhances nitric oxide bioavailability and ameliorates organ oxidative stress in transgenic-knockout sickle mice. Am. J. Physiol. Regul. Integr. Comp. Physiol..

[52] Zuckerman S.H., Bryan N. (1996). Inhibition of LDL oxidation and myeloperoxide dependent tyrosyl radical formation by the selective estrogen receptor modulator raloxifene (LY139481 HCL). Atherosclerosis.

